# SUPPORTING A MOTHER WITH DEMENTIA WHO MISTREATED ME: CAREGIVING EXPERIENCES OF STRAINED MOTHER-DAUGHTER DYADS

**DOI:** 10.1093/geroni/igad104.3734

**Published:** 2023-12-21

**Authors:** Mary Nemec, Laura Girling

**Affiliations:** Towson, Towson, Maryland, United States; Towson, Towson, Maryland, United States

## Abstract

Providing care to an aging parent is a normative expectation within most modern societies. Research steadily demonstrates that around two-thirds of caregivers are women, and over one-third of dementia caregivers are adult daughters. Often there is an assumption of natural attachment between mother and daughter—unconditional loyalty and permanent availability. However, overlooked in the literature is the notion that some adult daughters struggle with caregiving decisions regarding a parent with whom they have been maltreated. To date, little research has focused on dementia caregiving dynamics within strained mother-daughter dyads. To address this knowledge gap, data were drawn from the Aging at Home Alone with Alzheimer’s Disease study, an interview-based protocol with a purported sample of 120 community dwelling persons with dementia and their study partner. Thematic analyses were conducted on the subsample of daughters and their mothers with dementia (n=10) who reported a history of maltreatment (e.g., childhood neglect, childhood abuse). Thematic analyses were conducted on all case material for the subsample (e.g., interview data, case notes). Findings indicate four overarching themes: 1. Begrudging caregiving, 2. Fragmentary support, 3. Resurfacing childhood trauma, and 4. Post-traumatic growth and forgiveness. These themes stress the importance of how childhood maltreatment can shape emotional bonds early in life and affect dementia caregiving trajectories. Findings will be discussed in relation to support recommendations for caregiving dyads with a history of parental maltreatment.

